# Design and production of hybrid nanoparticles with polymeric-lipid shell–core structures: conventional and next-generation approaches

**DOI:** 10.1039/c8ra07069e

**Published:** 2018-10-09

**Authors:** Sabrina Bochicchio, Annalisa Dalmoro, Paolo Bertoncin, Gaetano Lamberti, Rouslan I. Moustafine, Anna Angela Barba

**Affiliations:** Dipartimento di Farmacia, Università degli Studi di Salerno Via Giovanni Paolo II, 132 84084 Fisciano SA Italy aabarba@unisa.it +39 089969240; Eng4Life Srl, Spin-off Accademico Via Fiorentino, 32, 83100 Avellino Italy; Dipartimento di Scienze della Vita, Centro Microscopia Elettronica, Università degli Studi di Trieste Via Fleming 31, A/B 34127 Trieste Italy; Dipartimento di Ingegneria Industriale, Università degli Studi di Salerno Via Giovanni Paolo II, 132 84084 Fisciano SA Italy; Department of Pharmaceutical, Analytical and Toxicological Chemistry, Kazan State Medical University Butlerov Street 49 420012 Kazan Russian Federation

## Abstract

Liposomes constitute a class of prominent drug delivery systems due their cell-mimetic behaviour. Despite their high biocompatibility, biodegradability and low intrinsic toxicity, their poor stability in biological fluids as well as in stock conditions (high tendency to degrade or aggregate) have led to new approaches for liposome stabilization (*e.g.*, surface covering with polymers). Here, liposomes were enwrapped by the natural biocompatible polymer chitosan to achieve stable shell–core nanostructures. Covered nanoliposomes were produced using an innovative continuous method based on microfluidic principles. The produced hybrid polymeric-lipid nanoparticles were characterized in terms of structural properties, size and stability. Moreover, phenomenological aspects in formation of nanoliposomal vesicles and chitosan layering, product quality (structure, size) and manufacturing yield related to this novel method were compared with those of the conventional dropwise method and the obtained products. The proposed simil-microfluidic method led to the production of stable and completely chitosan-covered liposomes with a shell–core nanostructure that avoided the disadvantages inherent in the conventional method (which are time-consuming and/or require bulky and more expensive equipment).

## Abstract 2

An innovative, simil-microfluidic, nanoliposome-covering method operating continuously with massive production yield overcoming the disadvantages of conventional methods is proposed.

## Graphical abstract

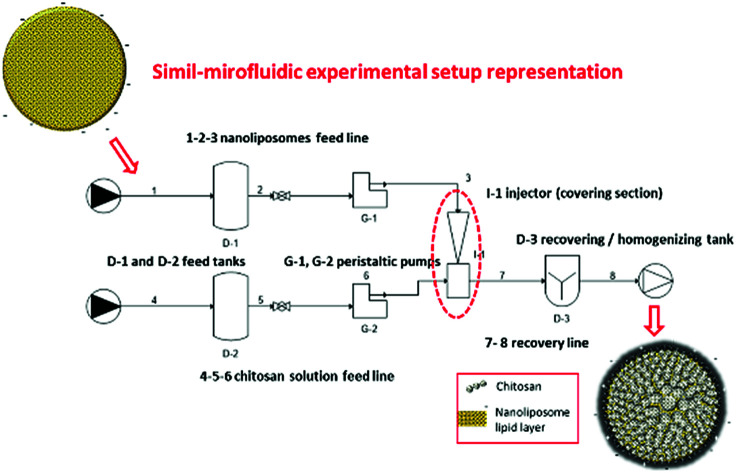

## 1. Introduction

The use of liposomes as pharmaceutical dosage systems, for encapsulating active molecules and releasing them in a controlled manner, represents an innovative and promising technology studied increasingly by the scientific community.^1–3^

The high level of biocompatibility, biodegradability, low intrinsic toxicity and immunogenicity makes liposomes sustainable materials that can increase the therapeutic index.^4^ Indeed, due to their cell-mimetic behaviour and the possibility of having nanoscale dimensions, liposomes present many advantages in drug delivery applications, and solve the major drawbacks of bioactive compounds: low stability, limited membrane permeability, short half-life and low bioavailability. Moreover, liposomes are versatile systems able to incorporate hydrophilic and hydrophobic molecules: hydrophilic drugs can be encapsulated in the interior aqueous compartment whereas lipophilic drugs can be incorporated into the liposome bilayer.^1,5–8^

Despite the advantages described above, the poor stability of these carriers into biological fluids as well as during storage (high tendency to degrade or aggregate), have led to the development (in tandem with recent advancements in nanotechnologies) of new approaches for liposome stabilization^9^ such as surface their covering with polymers.^10–12^

Different kinds of polymers (*e.g.* polyethylene glycol, PEG) have been used for the outer-surface modification of liposomes to extend their lifespan *in vivo*.^13^ Poly(ethylene oxide) (PEO) and poly(propylene oxide) (PPO) triblock copolymers with a high degree of hydrophilicity effectively protect liposomes from peroxidation, which can induce severe biomembrane dysfunctions at the cellular level and alter the chemical structures of polyunsaturated lipids at the molecular level.^14^ Liposomal vesicles have also been complexed with different pH-responsive copolymers, such as a hydrophobically modified copolymers of *N*-isopropylacrylamide, *N*-glycidylacrylamide, and *N*-octadecylacrylamide, to obtain pH-sensitive systems with stability >90 days,^15^ or randomly alkylated copolymers of *N*-isopropylacrylamide, methacrylic acid and *N*-vinyl-2-pyrrolidone for the production of stable complexes that slightly increased the circulation time of liposomes following intravenous administration.^16^ Moreover, with regard to covering applications, particular attention has been focused on chitosan.^17^ Chitosan is a biocompatible and biodegradable hydrophilic polymer which, due to its low toxicity, bio-adhesive and permeation-enhancing properties, has received much consideration as a liposome-complexing material for the release and targeting of drugs.^13,17,18^ Although chitosan-coated liposomes are starting to be produced for the delivery of several types of active molecules (*e.g.*, diclofenac sodium, leuprolide, superoxide dismutase, indomethacin, alkaloids, and other types of molecules with therapeutic properties^18–23^), the processes which lead to their production remain at the bench-scale, leading to small product volumes in output. Up to now, the methods usually employed for liposome covering by chitosan are based essentially on dropwise bulk methods^13,18,24–29^ such as the layer-by-layer (LbL) method.^30,31^ Some efforts have been made towards improvement of these conventional methods using, for example, a supercritical reverse-phase evaporation method,^32^ by exploiting complex and expensive apparatus.

Development of a novel liposome-covering method based on microfluidic principles with great potential for the industrial sector is presented here. This method is innovative in terms of reducing the cost impact on manufacturing due to a continuous production regime, massive production yield, ease of plant setup, and has been applied for covering nanoliposomes containing indomethacin as the active ingredient.^33^ In the present study, attention focused on the phenomenological aspects of formation of nanoliposomal vesicles and chitosan layering. Moreover, to emphasize the advantages of this innovative method, product quality (structure and size) and production yield were compared with the conventional dropwise method and related obtained products.

## 2. Experimental

### 2.1 Materials

l-α-Phosphatidylcholine (PC) from soybean, type II-S, 14–23% choline basis (CAS number 8002-43-5), cholesterol (CHO) (CAS number 57-88-5), chitosan (CAS number 9012-76-4; medium molecular weight and 75–85% deacetylated), ethanol of analytical grade (CAS number 64-17-5), glacial acetic acid (CAS number 64-19-7) and Triton X-100 (CAS number 9002-93-1) were purchased from Sigma–Aldrich (Milan, Italy). All the materials were used as acquired.

### 2.2. Methods

#### 2.2.1 Preparation of uncoated and chitosan-coated nanoliposomes

##### Production of uncoated-nanoliposomes

**Fig. 1:**
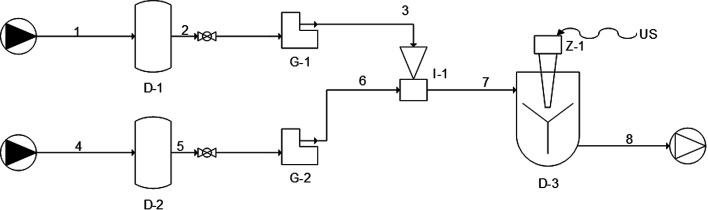
Piping representation for the experimental setup for the simil-microfluidic method: (1–2–3) lipids/ethanol feed line; (4–5–6) water feed line; (D-1 and D-2) feed tanks; (G-1 and G-2) peristaltic pumps; (I-1) injector (production section); (7–8) water/ethanol nanoliposomes suspension; (D-3) recovering/homogenizing tank.

Uncoated nanoliposomes were produced using the simil-microfluidic method by means of a semi-continuous setup developed previously, whose layout is described in ref. 34 and schematized in Fig. 1 by UNICHIM representation of piping. The values of the 10 : 1 volumetric flow rates ratio, which was defined as the hydration solution volumetric flow rate (*V*_hs_) to the lipid solution volumetric flow rate (*V*_ls_), and a lipid concentration of 5 mg ml^−1^ in the final hydro-alcoholic solution, were used for vesicle preparation. These conditions were chosen on the basis of previous work for the best results obtained for the dimensional features of liposomes after modulating the volumetric flow rates ratio (10 : 1, 15 : 1, 20 : 1 and 40 : 1 *V*_hs_/*V*_ls_) and lipid concentration in the hydro alcoholic solution (0.15, 1, 4 and 5 mg ml^−1^).^35^

Briefly, for production of uncoated nanoliposomes, a lipid/ethanol solution was prepared by dissolving 470 mg of PC and 94 mg of cholesterol in 10 ml of ethanol. Cholesterol, used at a 2.5 : 1 (mol mol^−1^) PC/CHOL ratio, was added to the formulation to stabilize vesicles. Deionized water (100 ml) was used as the hydration medium. The two feed solutions (lipids/ethanol and water, lines 1 and 4, respectively), taken from their feeding tanks (D-1 and D-2 in Fig. 1, respectively), were pushed through peristaltic pumps (G-1, line 2–3; and G-2, line 5–6) into the production section (I-1), where a hydro alcoholic solution containing vesicles based directly on nanometric size was formed (line 7). The suspension, recovered in a tank (D-3) was magnetically stirred for 1 h to promote ethanol evaporation. Then, part of the sample was subjected to characterization, and part of it was used for the chitosan-covering step (output line 8).

##### Chitosan-coated nanoliposomes prepared through simil-microfluidic method

Chitosan-coated nanoliposomes were prepared by the throughput simil-microfluidic method using the same experimental set-up as presented above (Fig. 1) using pipes 1–2–3 as the nanoliposome feed line and 4–5–6 as the chitosan solution feed line. Briefly, the previously prepared suspension of uncoated nanoliposomes (stored in tank D-1) and 0.01% w/v chitosan solution (stored in tank D-2) were pushed through peristaltic pumps (G-1 and G-2, respectively) at equal volumetric flow rates of 25 ml min^−1^ into the production section (I-1), where chitosan-coated liposomal vesicles were formed (line 7). Finally, the suspension was recovered in a tank (D-3, with US switch-off) and magnetically stirred for 1 h, and then particles were characterized (output line 8, products towards structural characterization).

##### Chitosan-coated nanoliposomes prepared through the dropwise method

**Fig. 2:**
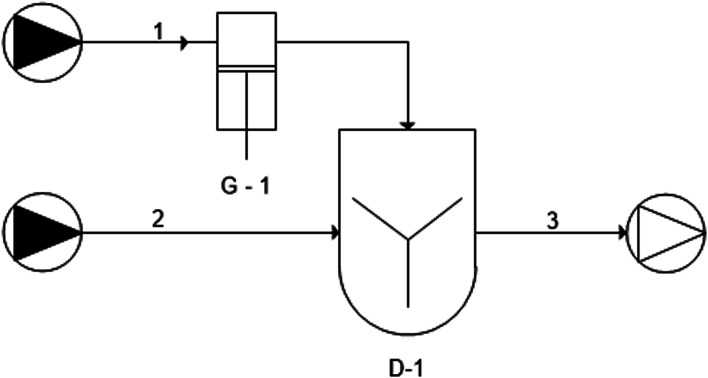
Piping representation for the experimental setup of the dropwise method: (1) chitosan solution feed line; (2) nanoliposome feed line; (3) suspension of chitosan-coated nanoliposomes; (G-1) syringe pump; (D-3) mixing tank.

To have a comparison in terms of production yield, product quality and setup scalability with the novel developed simil-microfluidic method, liposome coating was also done through the conventional dropwise method adopting the experiment setup schematized in Fig. 2 (by UNICHIM representation of piping). First, 10 mg of chitosan was dissolved in 10 ml of 0.5% (v/v) acetic acid and stirred for ∼90 min at room temperature until a clear stock solution was obtained, which was then diluted up to 0.01% w/v. Through use of a syringe pump, by means of a 0.66 ml min^−1^ volumetric flow rate (chitosan solution feed line 1), the solution (10 ml) was added dropwise to 10 ml of the liposomal suspension before preparation (nanoliposome feed line 2). The chitosan solution was added while stirring the unloaded liposomal suspension continuously at 200 rpm for 15 min at room temperature. The obtained solution was left stirring for an additional 1 h (D-1) and stored at 4 °C protected from light, then particles were characterized (output line 3).

#### 2.2.2 Characterization of uncoated and chitosan-coated nanoliposomes

##### Measurement of particle sizes and zeta potential

Dynamic light scattering (DLS) was done for dimensional characterization of uncoated and chitosan-coated vesicles using Zetasizer Nano ZS (Malvern Instruments). This system incorporates non-invasive backscatter (NIBS) optics to define the average hydrodynamic diameter (size) and the size distribution (polydispersity index (PDI)) of the vesicles; in particular, the particle size distribution plotted as the number of liposomes *versus* size. A detection angle of 173° able to measure the particle size of concentrated and turbid samples was used. The zeta potential (*ζ*) of uncoated and chitosan-coated liposomes was measured by photon correlation spectroscopy (PCS) using Zetasizer Nano ZS. All measurements were taken at room temperature using distilled water to disperse samples and were done in triplicate. Results are expressed as average values ± standard deviation (SD).

##### Morphology and structure

Morphological and structural characterization of uncoated and chitosan-coated liposomal vesicles was done using transmission electron microscopy (TEM) employing an EM 208 (Philips) system equipped with a camera (Quemesa, Olympus Soft Imaging Solutions). For this analysis, samples were diluted 1 : 1 with distilled water, then deposited on a Formvar/carbon support film on a specimen grid (Electron Microscopy Sciences). After air-drying for 5 min, the sample was negatively stained with 1% (w/v) of uranyl acetate solution for 10 min.

##### Stability tests: turbidity measurements and storage assessments

Turbidity measurements were done by solubilizing samples of liposomes and chitosan-coated liposomes by adding to the suspensions, in a continuous manner, fixed volumes of Triton X100 (from 0% to 5.7% v/v). After addition of each aliquot of surfactant into 2 ml cuvette containing the sample of interest, the suspensions were monitored by measuring their nephelometric turbidity units (NTU) by means of a turbidimeter (PCE-TUM 20, PCE Instruments). The NTU *versus* detergent concentration was noted until the sample of uncoated liposomes was solubilized entirely. All measurements were made in triplicate. Results are expressed as average values ± SD.

Stability studies (storage assessments) *in vitro* were undertaken on different lots of uncoated and chitosan-coated liposomes (produced by means of the dropwise method and simil-microfluidic method). The three types of lots, dispersed in distilled water, were sealed in 50 ml tubes and stored at 4 °C for 6 weeks. During this period, at given times, samples were inspected for coalescence by turbidity, size and zeta-potential measurements.

## 3. Results and discussion

### 3.1 Phenomenological aspects, formulative and fluidodynamic issues

As described in ref. 34, liposome formation through the simil-microfluidic method is governed by the molecular diffusion between lipids (which are solubilized in the organic phase) and water (which simultaneously diffuses into organic solvent to reduce its concentration below the critical value required for lipid solubilisation). In this way, liposomes based directly on nanometre size are formed. The simil-microfluidic method is based on the transposition at the millimetre scale of microfluidics-based methods. In this way, the method maintains the typical advantages of microfluidics by producing liposomes with precise control of size in an efficient and continuous manner and, simultaneously, overcomes its typical limitations (*i.e.*, increased costs for device microfabrication and low product volumes in output^36^).

Once nanoliposomes have been obtained, the chitosan-coating process (which is governed by the electrostatic interactions between the negatively charged liposome bilayer and the cationic polyelectrolyte) was started. In particular, the polyelectrolyte was adsorbed on the surface of the lipid particles, causing reversal of the net charge on the interface. Stable chitosan-coated liposomes are formed only if the chitosan concentration is below the saturation concentration (*C*_sat_) if all the polyelectrolytes added to the system are adsorbed to the surfaces of liposomes. If liposomes are completely saturated with chitosan, any extra amount of polymer added to the system will remain free in the suspension, generating a reduction in attraction between coated liposomes and, thus, their flocculation.^37^ Here, preliminary tests showed that chitosan concentrations higher than that used (0.01% w/v) caused flocculation of coated liposomes with both covering methods (simil-microfluidic and dropwise) because these concentrations were higher than the *C*_sat_ calculated for this system by the equation described in:^37^1
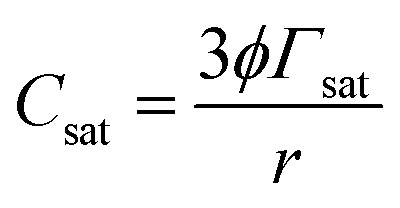
where *ϕ* is the volume fraction of the liposomes (approximated to the mass fraction of lipids and equal to 0.0026), *Γ*_sat_ is the surface load of the polyelectrolyte at saturation calculated as *Γ*_sat_ ≈ *M*/(*N*_A_π*r*_PE_^2^), where *N*_A_ is the Avogadro number, *M* and *r*_PE_ are, respectively, the molecular weight (250 kg mol^−1^) and effective radius of molecules of chitosan (estimated as 6 × 10^−8^ m from ref. 38), *r* is the volume-surface mean radius of liposomes (6 × 10^−8^ m, the mean diameter by DLS from this work as reported below).

From a phenomenological viewpoint, the mechanism of interaction between liposomes and chitosan was different for the two methods. In the dropwise coating method, initially just one drop of chitosan solution was added to the entire volume (bulk preparation) of liposome suspension. This led to the probable formation of coacervates between lipid and cationic chitosan molecules rather than the wrapping of chitosan around liposomes.^27^ Conversely, when additional chitosan solution drops were poured in, they can come into contact with already coated liposomes. This excess of chitosan concentration can cause the formation of agglomerates. The negative effects related to the variability of the chitosan concentration during dropwise coating were circumvented using the developed simil-microfluidic method. The latter allowed a continuous and uniform interaction between equal volumes of liposome solution and chitosan solution, leading to the production of more stable and completely covered liposomal systems.

Assessments of the fluid dynamics of feeds have a key role in formation of liposomal vesicles in the simil-microfluidic method. Thus, the flow regimen was evaluated for preparation of uncoated liposomes and chitosan-coated liposomes to verify their laminar features (typical of microfluidic systems) by checking that all Hagen–Poiseuille assumptions had been satisfied.^39^ First, the Reynolds number was calculated:2
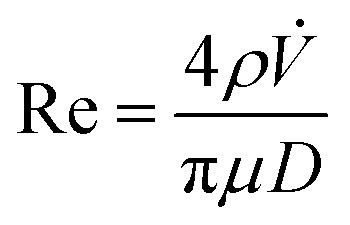
where *ρ* is the fluid density, *V̇* is the volumetric flow rate of the fluid, *μ* is its dynamic viscosity, π is the mathematical constant 3.14, and *D* is the tube diameter. For determination of the fluid dynamic conditions, some approximations were made. That is, in the preparation of uncoated liposomes, the organic phase was referred only to ethanol, the polar phase only to water, and also the liposomal suspension was referred to a water solution (being the alcohol used only at 9% v/v); in the liposome-coating method, all the chemical–physical and transport properties of the three solutions (liposomes suspension, chitosan solution, and coated-liposomes suspension) were approximated to those of water (Table 1). Thus, for production of uncoated liposomes, the polar and organic phases exhibited a laminar flow regimen in the feeding section, likewise the hydro-alcohol phase in the production section. In the same way, in the liposome-coating process, the liposome suspension and chitosan solution in the feeding section, together with the coated-liposome suspension in the production section, showed a Reynolds number < 2100. Moreover, the other Hagen–Poiseuille assumptions were also satisfied. In effect, both processes were carried out at constant temperature and pressure, thus the fluids were considered incompressible and the flow was considered to be steady, except for the startup moment (due to the volumetric flow rates being kept constant). All involved fluids was considered to be Newtonian because the behaviour of the (coated and uncoated) liposome suspensions had been approximated reasonably to that of water due to the concentration used.

**Table 1:** Parameters for calculations of the Reynolds number and entrance length

	Uncoated liposome production			Coated liposome production		
	Feeding inner phase	Feeding outer phase	Production phase	Feeding inner phase	Feeding outer phase	Production phase
Tube diameter, m	1.6 × 10^−3^	5 × 10^−3^	3 × 10^−3^	1.6 × 10^−3^	5 × 10^−3^	3 × 10^−3^
Flow rate, m^3^ s^−1^	7.5 × 10^−8^	7.5 × 10^−7^	8.2 × 10^−7^	4.2 × 10^−7^	4.2 × 10^−7^	8.4 × 10^−7^
Fluid type	Ethanol lipid solution	Hydration solution	Liposomes suspension	Liposomes suspension	Chitosan solution	Coated liposomes suspension
Fluid approximation	Ethanol	Water	Water	Water	Water	Water
Density, kg m^−3^	789	1003	1003	1003	1003	1003
Viscosity, Pa s	1.2 × 10^−3^	1 × 10^−3^	1 × 10^−3^	1 × 10^−3^	1 × 10^−3^	1 × 10^−3^
**Re**	**39**	**192**	**351**	**333**	**106**	**355**
***L***_**e**_ **, m**	**—**	**—**	**0.037**			**0.037**

Finally, the piping length in which the two phases diffuse was checked to be longer than the “entrance length” needed to build-up the parabolic profile. Thus, to neglect the end effects and thus fulfil another Hagen–Poiseuille assumption, the entrance length (*L*_e_) after the entrance of the inner-phase tube in that of the outer-phase tube was calculated to be *L*_e_ = 0.035*D*Re, where Re and *D* are parameters of the production phase tube. A 37 mm entrance length was found for both processes (Table 1), lower than that of the pipe (150 mm), where interdiffusion phenomena occurred.

This novel process, based on microfluidic principles, led to massive production of particles. It was possible to prepare 1 L of a suspension of chitosan-coated liposomes in just 20 min, the same time required to manufacture 20 ml of product with the classical dropwise method^20^ (though some scholars have reported even higher production times (2 h) for preparation of that volume^18^). In principle, the simil-microfluidic method can be continuous; this is an important innovation towards applications on the industrial scale.

### 3.2 Characterization of uncoated and chitosan-coated nanoliposomes

#### 3.2.1 Particle sizes and zeta potential

**Fig. 3:**
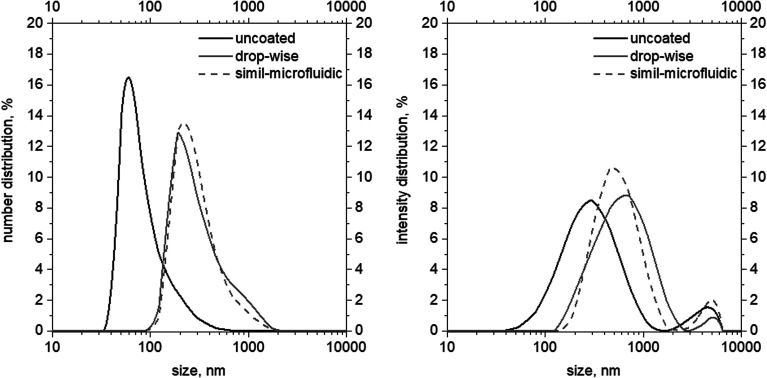
Size (left, numerical distributions) and *Z*-average (right, intensity distribution) distributions of uncoated, dropwise-coated, and simil-microfluidic-coated nanoliposomes.

The particle size, PDI and zeta potential of liposomes, chitosan-coated liposomes by the dropwise method, and chitosan-coated liposomes through the simil-microfluidic method are displayed in Table 2 and Fig. 3.

**Table 2:** Size, PDI, and zeta potential of uncoated and chitosan-coated nanoliposomes. Results are the average of three determinations ± SD

	Size, nm (number distribution)	*Z*-Average, nm	PDI	Zeta potential, mV
Uncoated liposomes	60 ± 30	260 ± 1	0.40 ± 0.01	−40 ± 1
Chitosan coated liposomes, drop-wise method	210 ± 20	550 ± 8	0.30 ± 0.02	−20.0 ± 0.5
Chitosan coated liposomes, simil-microfluidic method	260 ± 40	500 ± 10	0.30 ± 0.03	−20.0 ± 0.9

Observation of data on numerical and intensity distributions (on left and right in Fig. 3, respectively) of uncoated, dropwise-coated, and simil-microfluidic-coated nanoliposomes revealed that chitosan coverage produced larger particles with similar features for both types of coverage method. In particular, the average number size and *Z*-average (reported in Table 2) were the average values of the Gaussian curve-fitting number and intensity distribution, respectively (the number size distribution gives information about the number of particles with a specific size range, and the intensity distribution is proportional to particle weight). Intensity distributions shifted to higher abscissa values (size) (Fig. 3). In particular, here the intensity distributions had two peaks, the higher one (which was representative of the *Z*-average values) and a shorter one (representative of a small portion of aggregates). The latter were very limited, as confirmed by the number distribution, where the second peak disappeared.

Nanometric uncoated lipid vesicles with an average diameter size of ∼60 nm and a *Z*-average of ∼260 nm were obtained; the chitosan coating of nanoliposomes led to an increase in lipid-vesicle size independent from the used coating method without a significant impact on the vesicle size distribution (PDI). In particular, chitosan-coated nanovesicles with a dimension in the range 210–260 nm (values coming from a number distribution) and with a *Z*-average range of 500–550 nm were obtained after the coating step (Table 2).

The zeta potential is a measure of the surface electrical charge of the produced vesicles. Thus, it is a crucial index of the stability of suspensions, and was also measured. Indeed, to avoid aggregation (which results in precipitation), liposomes should have a |*ζ*| > 30 mV; if the zeta potential drops below this value, the system becomes unstable and begins to form larger complexes as attractive forces prevail.^40^ Here, stable vesicles were produced with a negative zeta potential (−40 ± 1 mV) due to the polyunsaturated fatty acids (linoleic and oleic acids) in phosphatidylcholine vesicles. Starting from stable lipid vesicles (*ζ* > −30 mV), thereby avoiding aggregates, is an essential prerequisite for the success of the next coating step.

In that regard, the electrostatic interactions between the negative charges of the liposomal bilayer and cationic chitosan solution tended to increase the zeta potential of the coated particles, whose value became less negative, thereby confirming successful coverage of vesicles. Interestingly, using the simil-microfluidic method for production of chitosan-coated nanoliposomes, and maintaining their unchanged chemical composition, particles with a zeta potential that was less negative (−20 mV) as that produced through the dropwise method (−20 mV), were obtained (Table 2). This suggests that a more effective coating process is achievable through the simil-microfluidic method.

#### 3.2.2 Morphology and structure

**Fig. 4:**
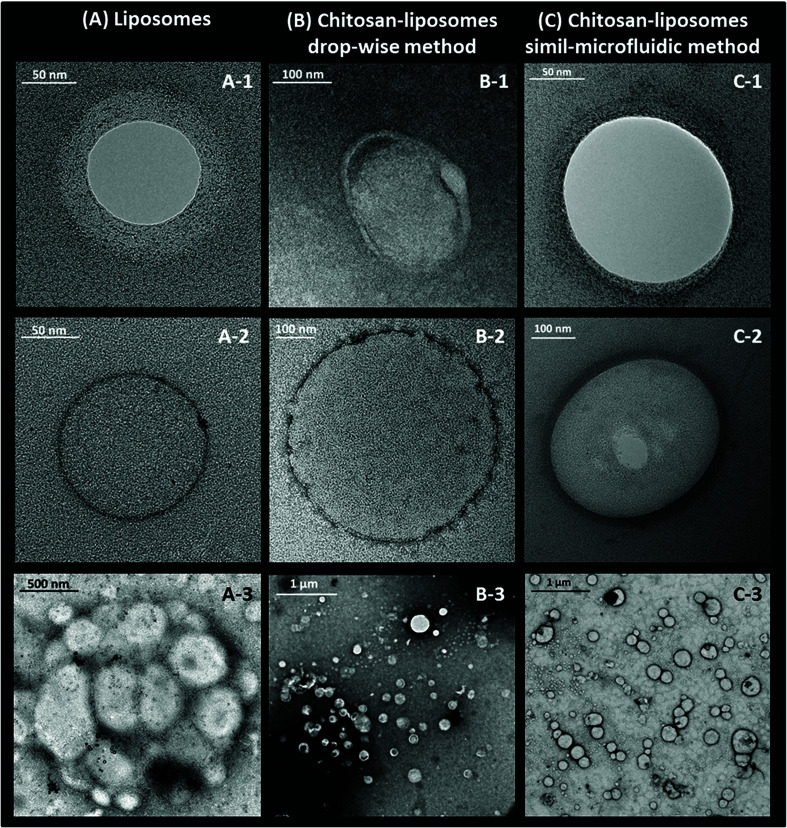
Transmission electron micrographs of (A) nanoliposomes, (B) chitosan-coated nanoliposomes prepared through the dropwise method and (C) chitosan-coated nanoliposomes prepared through the simil-microfluidic method.

TEM images are shown in Fig. 4. They show the morphological, structural and aggregation tendency of uncoated (Fig. 4(A1–A3)) and coated (Fig. 4(B1–B3 and C1–C3)) nanoliposomes.

**Fig. 5:**
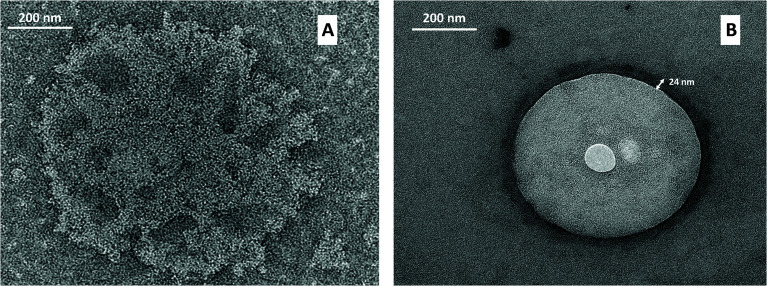
Detailed TEMs of chitosan-coated nanoliposomes achieved by the dropwise method (A) and simil-microfluidic method (B).

As shown in Fig. 4, morphological studies on particle shape indicated that spherical nanoliposomes (Fig. 4(A)) were obtained through the simil-microfluidic method, and that chitosan coating had not altered the vesicle shape significantly (Fig. 4(B and C)). The chitosan layer surrounding nanoliposomes was clearly visualized on the surface of vesicles obtained through both covering methods. However, the coating obtained with the simil-microfluidic method gave vesicles with a thick and smooth polymeric surface (clearly visible in Fig. 4(C1 and C2)), chitosan-nanoliposomes produced by the dropwise method showed a very thin coating which was rough and uneven (Fig. 4(B1 and B2)). Reasonable interpretations can be found in the electrostatic interactions that take place during the two coating processes (detailed TEM of chitosan-coated liposomes achieved by dropwise and simil-microfluidic methods are reported in Fig. 5(A and B), respectively). In the simil-microfluidic method, the contact between the negatively charged surface of liposomes and the cationic chitosan was uniform and continuous. Indeed, during the covering process, at each time the liposome suspension interacted with an equal volume of chitosan solution, so the chitosan concentration was kept constant at 0.005% w/v for all the covering time (because the nanoliposome suspension and chitosan solution met at the same flow rate). At these low concentrations, chitosan is a linear polysaccharide with extended polymer chains. These are adsorbed flat onto liposomes, thereby maintaining the integrity of lipid bilayers and improving their physicochemical properties, including a decrease in membrane fluidity, enhancement of the longitudinal order of the bilayer (with a layer coating thickness of 21 ± 5 nm, as visible in Fig. 5(B) for the simil-microfluidic method), and inhibition of lipid oxidation.^41^ Instead, in the dropwise method, one drop of chitosan solution falling into the nanoliposome suspension covers just the vesicles present in that area and, although the sample is magnetically stirred, chitosan is dispersed less uniformly in the liposome suspension, causing less coating of particles. In some cases, the chitosan can already meet coated liposomes, thus a local excess of chitosan causes coil formation and results in the formation of “edge defects” in the lipid bilayer (as visible in Fig. 5(B) for the dropwise method), which can cause destabilization of membrane dynamics and structural properties.

Apart from the method used, it is evident that the chitosan-coating strategy can drastically reduce the tendency of nanoliposomes to coalesce and aggregate. The cluster of liposomes shown in Fig. 4(A3) disappeared after the covering steps (Fig. 4(B3 and C3)), thereby confirming that the coating of nanoliposomes with a chitosan layer resulted in an increase in particle stability. This result was also confirmed by the zeta potential and the turbidity monitoring, as described below.

#### 3.2.3 Stability tests

**Fig. 6:**
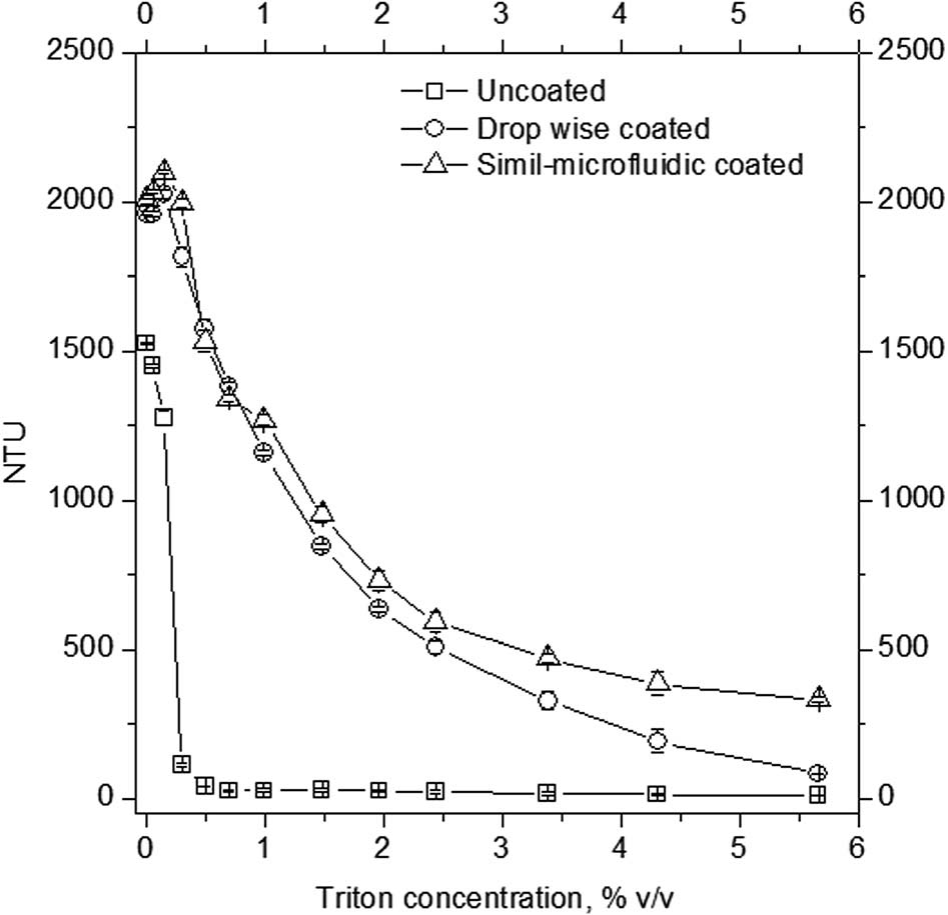
Turbidity of suspensions of uncoated nanoliposomes (open squares), chitosan-coated nanoliposomes produced through the dropwise method (open circles), and chitosan-coated nanoliposomes produced through the simil-microfluidic method (open triangles) at increasing concentrations of the detergent Triton X100.

**Fig. 7:**
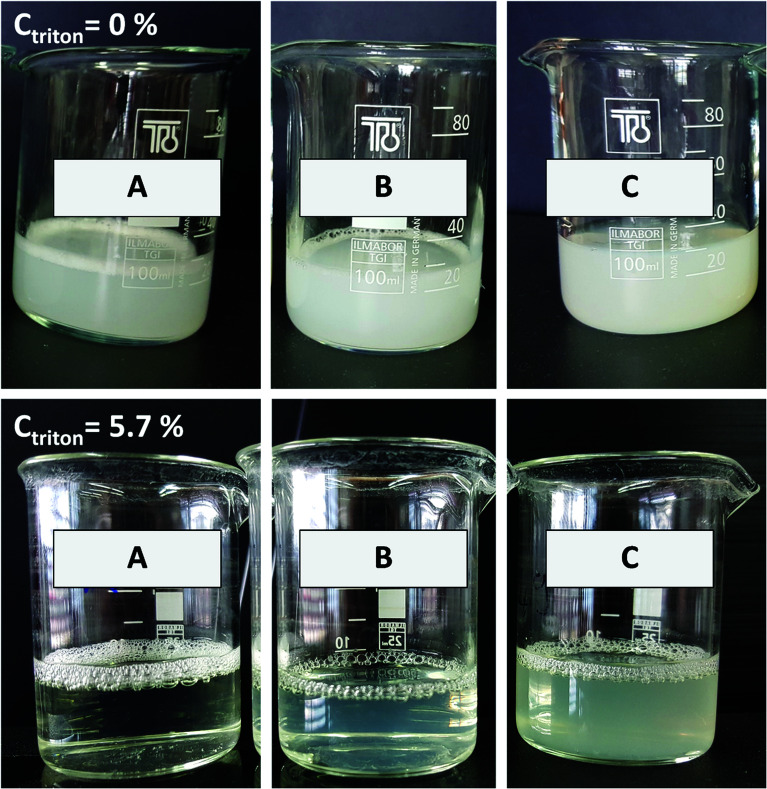
Photographs of particles before (up) and after treatment with Triton X100 (5.7% v/v) (down): (A) uncoated nanoliposomes; (B) chitosan-coated nanoliposomes produced through the dropwise method; (C) chitosan-coated nanoliposomes produced through the simil-microfluidic method.

Liposomal solubilisation can be used as a molecular tool for studies of vesicle stability and chitosan–lipid interactions. As described by Helenius and Simons,^42^ if a detergent is added to a suspension of liposomes, part of it interacts with the lipid bilayer and part of it remains free in the solution, leading to the formation of different complexes, such as micelles incorporating phospholipids or phospholipid bilayers containing detergent, or other mixed structures;^42^ this leads to a change in the physical properties of the bilayer and, thus, to its solubilisation. In this work, the resistance of particles to these physical changes were studied by monitoring the turbidity of the samples after detergent addition. As shown in Fig. 6, at equal amount of Triton X100 added to the samples led to an evident difference in solubilisation between uncoated and chitosan-coated nanovesicles. First, before Triton X100 addition to the suspensions, the uncoated vesicles presented a turbidity lower (∼24% less) than those characterizing the chitosan-coated particles. When Triton X100 (0.3% v/v) was added to the suspensions, an abrupt decrease in NTU was observed for uncoated liposomes, which were almost entirely dissolved (about ∼93% of the sample was solubilized). Instead, at the same detergent concentration, ∼0.6% of the sample was dissolved if coating was done through the simil-microfluidic method and ∼7% of the vesicles were solubilized if they were covered by means of the dropwise method. Starting from Triton X100 at 0.3% v/v up to the last concentration used (5.7% v/v), while the trend of uncoated vesicles did not change in a relevant manner (the sample was totally solubilized), solubilization of the particles produced by the simil-microfluidic method appeared to be less than that observed for the sample obtained through the dropwise method. Indeed, at the final detergent concentration used, uncoated vesicles and those coated by the dropwise method were all solubilized, whereas the 17% of vesicles covered by the simil-microfluidic method had not yet dissolved. This result showed that the simil-microfluidic method, which gave a more uniform covering, conferred major stability to the particle structure as confirmed by photographic images taken for uncoated and chitosan-coated liposomes before and after treatment with the maximum Triton X100 concentration (5.7% v/v) (Fig. 7). In particular, at the final non-ionic surfactant concentration, the opacity increased gradually from sample A (uncoated) to B (coated using the dropwise method) to C (coated using the simil-microfluidic method).

**Fig. 8:**
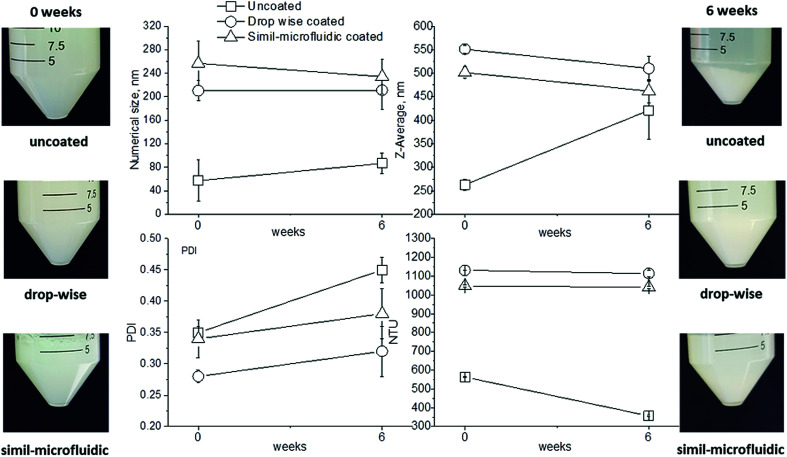
Variation of appearance (photographs), number size, *Z*-average, PDI and turbidimetry measurements of suspensions of uncoated nanoliposomes, chitosan-coated nanoliposomes produced through the dropwise method, and chitosan-coated nanoliposomes produced through the simil-microfluidic method, from 0 to 6 weeks of storage.

The ability of chitosan to stabilize the produced nanostructures was also observed during storage stability tests (Table 3). The zeta potential of uncoated nanoliposomes during 6 weeks of storage fluctuated and was unpredictable. It increased from about −40 mV at time 0 to −20 mV after 1 week, decreased to about −40 mV after 3 weeks and, finally, it increased again to −20 mV after 6 weeks. These data showed a lack of stability for uncoated liposomes, which tended to aggregate and disaggregate continuously during storage, thereby changing their superficial charge. Conversely, coated nanoliposomes, both with the dropwise method and simil-microfluidic method, did not show significant variation of zeta potential during 6 weeks of storage (measurements are reported in Table 3), suggesting the efficacy of chitosan coating in stabilizing liposomes against aggregation. These results (*i.e.*, aggregation for uncoated nanoliposomes and good dispersion for coated nanoliposomes during storage) were confirmed by the time evolution of size (number and *Z*-average), PDI and the turbidity of analyzed samples (Fig. 8). From visual observations it was clear that after 6 weeks the suspension with uncoated liposomes was characterized by sedimentation, which was not seen in the suspension of coated nanoliposomes. Aggregation during this time for uncoated nanoliposomes was also confirmed by an increase in size (especially in *Z*-average and PDI), and by a decrease in the turbidity of samples due to sedimentation. Conversely, significant changes of these values were not recorded for chitosan-coated nanoliposomes.

**Table 3:** Zeta potential of uncoated and chitosan-coated nanovesicles during studies of storage stability

Zeta potential, mV	0 weeks	1 week	3 weeks	6 weeks
Uncoated liposomes	−40 ± 1	−20.0 ± 0.9	−40 ± 1	−30 ± 2
Chitosan coated liposomes, drop wise method	−20.0 ± 0.5	−20.0 ± 0.5	−20 ± 2	−20 ± 1
Chitosan coated liposomes, simil-microfluidic method	−20.0 ± 0.8	−20.0 ± 0.1	−20 ± 2	−20 ± 1

## 4. Conclusions

Liposomes were produced by the simil-microfluidic method at the nanoscale by a continuous and rapid procedure. Adopting the same experimental setup, a stream of chitosan solution was used to wrap nanoliposomes to obtain hybrid polymeric-lipid nanostructures with a shell–core architecture. DLS and TEM confirmed the nano-size of the products (vesicles with an average diameter of ∼260 nm). Moreover, TEM images emphasized the success of the covering process.

Stability tests undertaken to investigate aggregation/denaturation and conducted by turbidity and zeta-potential measurements and imaging confirmed the perfect suspension in time of the covered nanostructures. Uncovered liposomes and covered liposomes obtained by the dropwise method were also obtained at the nanoscale (average diameter of 60 nm and 210 nm, respectively) but showed poor stability due to the absence/partial failure of chitosan wrapping.

Our novel method could achieve massive production of particles. For example, we could prepare 1 L of chitosan-coated liposomes in just 20 min; with the classical dropwise method, in 20 minutes, only few millilitres could be processed. The method described here can drive a continuous process with high production yield and tailored properties of final products.

## Conflicts of interest

There are no conflicts of interest to declare.

## Supplementary Material
